# Clinical Treatises on the Pathology and Therapy of Disorders of Metabolism and Nutrition

**Published:** 1903-09

**Authors:** 


**Clinical Treatises on the Pathology and Therapy of Disorders of Metabolism and Nutrition.** By Prof. Dr. CARL VON NOORDEN. Authorised American Edition. Translated under the direction of BOARDMAN REED, M.D. Part III.—*Membranous Catarrh of the Intestines* (*Colica Mucosa*). Pp. 64. New York: E. B. Treat and Company. 1903.

This little book gives a concise account of the curious and rather obscure affection known as Membranous Catarrh of the Intestines. The term colitis cannot be used for all cases, for though "colica mucosa" may be one of the signs of genuine enteritis, it may also occur without any anatomical lesion of the intestinal mucous membrane. Obstinate constipation is an important factor, but does not play the chief part, in producing it. The author considers that in addition to general hysteria or neurasthenia there is a morbid change in the secretion of mucus by the intestine; that is, an alteration in the nervous mechanism that regulates its secretion. With regard to treatment the directions are clear and definite. Prof. von Noorden, after enforcing the necessity of rest and of emptying the bowels by enemata of hot water followed by oil as a commencement of treatment, lays stress upon the paramount importance of a suitable diet; and his experience has taught him that in place of giving a bland diet leaving little indigestible residue, one that is very coarse and contains a large amount of indigestible material, such as whole meal bread, vegetables containing much cellulose, fruit with small seeds and thick skins, in addition to large quantities of fat, should be employed. The object of this diet is to produce soft and massive stools. Massage of the large intestine is used as an adjuvant, and the object of the cure is not attained until the bowels are regularly and naturally moved without medicine. Then "the disease 'colica mucosa'" may be considered cured, and will never return."

